# The CYP1A2 -163C>A polymorphism does not alter the effects of caffeine on basketball performance

**DOI:** 10.1371/journal.pone.0195943

**Published:** 2018-04-18

**Authors:** Carlos Puente, Javier Abián-Vicén, Juan Del Coso, Beatriz Lara, Juan José Salinero

**Affiliations:** 1 Exercise Physiology Laboratory, Camilo José Cela University, Madrid, Spain; 2 Performance and Sport Rehabilitation Laboratory, University of Castilla La Mancha, Toledo, Spain; Laval University, CANADA

## Abstract

**Purpose:**

The aim of this investigation was to analyze the influence of the genetic variations of the -163C>A polymorphism of the CYP1A2 gene on the ergogenic effects of caffeine in elite basketball players.

**Methods:**

Nineteen elite basketball players (10 men and 9 women) ingested 3 mg⋅kg^-1^ of caffeine or a placebo 60 min before performing 10 repetitions of the following series: the Abalakov jump test followed by the Change-of-Direction and Acceleration Test (CODAT). The players then competed in a 20-min simulated basketball game. Self-perceived performance and side effects were recorded by questionnaires after the trials. The effects of caffeine on basketball performance were established according to players’ CYP1A2 genotype (rs762551): AA homozygotes (n = 10) and C-allele carriers (n = 9).

**Results:**

In the 10 repetitions, caffeine increased Abalakov jump height by a mean of 2.9±3.6% in AA homozygotes (*p* = 0.03) while this effect did not reach statistical significance for C-allele carriers (2.3 ± 6.8%; *p* = 0.33). Caffeine did not affect sprint time in the CODAT test in either genotype group but it increased the number of impacts performed during the simulated game in both AA homozygotes (4.1 ± 5.3%; *p* = 0.02) and C-allele carriers (3.3 ± 3.2%; *p* = 0.01). During the 24 h following the test, AA homozygotes tended to experience increased insomnia with caffeine while C-allele carriers did not present this effect. The remaining variables were unaffected by the genotype.

**Conclusion:**

The CYP1A2 -163C>A polymorphism minimally altered the ergogenicity derived from the consumption of a moderate dose of caffeine in elite basketball players.

## Introduction

Caffeine or caffeinated products are commonly used as ergogenic aids in sports because of their efficacy to increase physical performance in both team and individual sports with different physiological demands [1–6]. The popularity of caffeine in sports is due to its measurable ergogenic effects even when consumed in low-to-moderate doses (from 3 to 6 mg·kg^-1^; [7–10]) and its effectiveness seems to be related to the correct identification of the substance and or the perception of increased performance produced by the ingestion of caffeine [11, 12]. These notions are grounded on previous investigations that have reported the benefits of caffeine for physical performance as a group mean. However, a few investigations have shown that not all individuals experience enhanced physical performance after the ingestion of moderate doses of caffeine [6, 13, 14] which suggests that this stimulant might not be ergogenic for all athletes. Indeed, these studies have identified the presence of athletes that obtain minimal ergogenic effects or even slightly ergolytic effects after caffeine intake (e.g., “non-responders to caffeine” [15]).

To date, there is still no clear explanation for the lack of ergogenic effects after the acute of caffeine in some individuals, while factors such as training status, habitual daily caffeine intake/tolerance to caffeine and/or genotype variation have been proposed as probable modifying factors for the ergogenicity of caffeine [15]. Perhaps, the most tested research hypothesis for this topic has been the one that relates the inter-individual differences in the ergogenicity of caffeine and the genetic polymorphisms in cytochrome P450 proteins, the hepatic enzymes responsible for caffeine metabolism. Specifically, CYP1A2 is a drug-metabolizing enzyme with major relevance for caffeine metabolism because it catabolizes caffeine into paraxanthine and other dymethylxanthines [16]. A single nucleotide polymorphism (SNP) in the CYP1A2 gene (-163C>A; rs762551) is responsible for the ultra-fast CYP1A2*1F haplotype which confers a faster capacity to metabolize caffeine on AA homozygotes [17]. According to this notion, AA homozygotes could catabolize caffeine into paraxanthine faster than CA and CC individuals [18], which in turn could produce a higher clearance of this substance from the blood and reduce the ergogenic effects from caffeine ingestion in these individuals.

Previous investigations have been aimed at determining the influence of the -163C>A SNP on the ergogenic effects of caffeine [19–23], although the outcomes of this research are inconsistent. It has been found that C-allele carriers (CC homozygotes and CA heterozygotes) experienced a greater improvement with caffeine (6 mg·kg^-1^) than AA homozygotes during a 3 km cycling trial [20]. On the contrary, AA homozygotes had greater ergogenicity compared to C-allele carriers after ingesting caffeine (6 mg·kg^-1^) prior to a 40-km time trial [19]. It has even been found that the ergogenic effects of caffeine are unmodified by the -163C>A SNP because the change in performance induced by 3–4 mg·kg^-1^of caffeine was similar for AA homozygotes and C-allele carriers during a 15-min cycling test, [22] and during the Wingate test [23], with no changes in post-exercise heart rate variability between these two genotypes [21].

Thus, the aim of the present study was to analyze the influence of the genetic variations of the CYP1A2 gene (-163C>A) on the ergogenic effects derived from a moderate dose of caffeine in elite basketball players, to increase the available information about the mechanism(s) related to the inter-individual variability in the ergogenicity of caffeine.

## Methods

### Participants

Nineteen elite basketball players volunteered to participate in this study. The initial study sample included 10 semiprofessional male basketball players and 10 professional female basketball players but one female participant did not give her consent for the obtaining of the genetic information through blood or saliva samples. The results of caffeine intake on the performance tests, without the influence of the CYP1A2 gene, has been recently published elsewhere [10]. All participants had prior basketball experience of at least 10 years and had trained for approximately 2 h·day^-1^, 5 days·week^-1^ (including a weekly competition) during the previous year. Players had no previous history of cardiopulmonary diseases and were not taking medications or sympathetic stimulants during the duration of the investigation. Moreover, all of the subjects were non-smokers and light-caffeine consumers (<100 mg·day^-1^). All female players were always tested during the luteal phase.

### Ethics statement

Participants were fully informed of the experimental procedures and the benefits and risks associated to the experiment before giving their informed written consent to participate. The investigation was approved by the University Ethics Committee in accordance with the latest version of the Declaration of Helsinki.

### Pre-experimental procedures

One week before the experimental trials, participants underwent a physical examination to ensure that they were in good health. After that, players were nude weighed (±50 g, Radwag, Poland) to individualize caffeine doses. Afterwards, a venous blood sample (5 mL) was obtained from an antecubital vein and inserted into a tube with EDTA and refrigerated (4 °C). This sample was later analyzed to determine genetic variations in the 163C>A (rs762551) SNP using standard procedures [22]. On the same day, participants’ anthropometric characteristics were registered by an ISAK-certified anthropometrist and body fat percentage was calculated using six skin folds [24]. Participants were also encouraged by the researchers to abstain from caffeine in any form (e.g. cola, coffee, energy drinks, etc.) and alcohol for 48 h before testing -the abstention of caffeine was maintained during the whole experiment to reduce habituation to this substance-.

The day before each experimental trial, players refrained from strenuous exercise and adopted a similar diet and fluid intake regimen, mimicking their pre-competition routines. They were also instructed to have their habitual pre-competition meal at least three hours before the onset of the experimental trials, with proportions of 60/16/24% for carbohydrate/protein/fat (CESNID, Barcelona, Spain). These standardizations were reported to the technical staff of the basketball team to ensure compliance, and were confirmed with individualized dietary and training diaries.

### Experimental design

A case-control ecological experimental design was used for this investigation. Initially, the participants were divided into three groups, established according to their individual genetic profile in the single nucleotide polymorphism 163C>A of the CYP1A2 gene (e.g., AA, CA and CC groups). However, C-allele carriers (e.g., CA and CC) were clustered and considered as a single group due to a preliminary analysis that showed very similar phenotyping responses in these individual participants. Moreover, this same collapsing strategy has been previously suggested for this SNP [19, 21–23]. This study treated male and female basketball players as a single group in the statistical analysis because no sex interactions have been previously found in the use of caffeine in sports ([11, 25]; Table 1).

**Table 1:** Age and anthropometric characteristics for AA homozygotes (n = 10) and C-allele carriers (n = 9) in the -163C>A polymorphism of the CYP1A2 gene.

Variable	Men	Women	Total
**Age (years)**			
AA homozygotes	26.5 ± 2.4	27.0 ± 5.3	26.7 ± 3.5
C-allele carriers	27.8 ± 5.6	31.0 ± 6.7	29.4 ± 6.0
**Body height (cm)**			
AA homozygotes	199.0 ± 7.4	172.3 ± 12.1	187.6 ± 16.7
C-allele carriers	186.8 ± 5.1	178.8 ± 6.1	182.8 ± 6.7
**Body mass (kg)**			
AA homozygotes	93.3 ± 8.6	70.4 ± 23.4	83.5 ± 19.2
C-allele carriers	85.6 ± 17.7	71.1 ± 7.0	78.4 ± 14.7
**Body fatness (%)**			
AA homozygotes	13.3 ± 3.3	14.2 ± 5.1	13.7 ± 3.8
C-allele carriers	10.2 ± 2.1	16.5 ± 1.3	13.3 ± 3.7
**Body muscle (%)**			
AA homozygotes	50.0 ± 7.2	47.4 ± 3.0	48.5 ± 4.8
C-allele carriers	49.2 ± 1.5	46.7 ± 1.1	47.9 ± 1.8

### Experimental protocol

For this investigation, all the basketball players underwent the same testing under the same experimental conditions, participating in a double-blind, placebo-controlled randomized experiment. Each participant took part in two trials separated by one week and the testing was carried out at the same time of day (from 7 to 9 p.m.) and with the same ambient conditions (indoor facility at 18.5±0.8 °C dry temperature; 30.8±1.0 relative humidity). On one occasion, subjects took 3 mg of caffeine per kg of body mass (3 mg⋅kg^-1^; 99% purity, BulkPowders, UK) in an opaque capsule. On another occasion, players ingested an identical opaque capsule with the same color but filled with a placebo substance (cellulose). The capsule was ingested 60 min before the onset of testing to allow complete caffeine absorption [26]. The order of the experimental trials (e.g. caffeine or placebo) was randomized and counterbalanced. However, the order of the testing days was set so that all the players from the same team received the same treatment (caffeine or placebo) to facilitate the analysis of the game statistics related to each basketball team. The capsules were prepared by a researcher that did not take part in the experimental trials and an alphanumeric code was assigned to each day of testing to blind participants and investigators to the substance ingested by each team.

On the day of the experimental trial, players arrived at their habitual basketball court and ingested the capsule assigned for the trial with 250 mL of water. They wore the same clothes they usually use to compete in (T-shirt, shorts and basketball shoes) for both days. An accelerometer/heart rate device (SPI PRO X, GPSports^®^, Australia) and a heart rate monitor (Polar^®^ T31, Finland) were provided to each player to be inserted in a purpose-built harness. Afterwards, the basketball players performed a standardized warm-up for 30 min consisting of a continuous run, dynamic flexibility, dribbling and shooting exercises. Then, they performed 10 repetitions of a combination of jumps, sprints and free throws (2 min of recovery between repetitions). In each repetition, players performed an Abalakov jump (Optojump next; Microgate, Bolzano, Italy; DSD Sport System, Spain; [27]) and a Change-of-Direction and Acceleration Test (CODAT; [28]). There was no recovery time between the jump and the sprint, but a 14 s recovery time was set between repetitions. The CODAT was performed without (from 1^st^ to 5^th^ repetitions) and with the basketball ball (from 6^th^ to 10^th^ repetitions) to assess the effects of caffeine on running velocity with changes of directions with and without ball bounce.

After a 20-min recovery period, players participated in a simulated game played on an official basketball court. The game consisted of two halves of 10 minutes with a break of 2 minutes between them, following the rules of the International Basketball Federation (FIBA). Two professional referees made the decisions during the games. Each basketball team was composed of the same individuals on both days: one guard, two forwards and two centers. Changes among players and time-outs were not allowed. Players were required to play with an individual defense and to attack without closed and predetermined game systems during the whole match. Body impacts and heart rate were continuously monitored during the game using accelerometer/heart rate devices. When the game finished, participants filled out a questionnaire about their sensations of endurance, muscle power and overall perceived exertion (RPE) during the game. This questionnaire included a 1- to 10-point scale to individually assess each item (1 point meant the minimal amount of that item and 10 points meant the maximal amount of the item [11]). In addition, participants were provided with a survey to be filled out the following morning about gastrointestinal problems, insomnia, nervousness and other discomforts perceived during the hours after the game. This questionnaire included seven items on a yes/no scale and has been previously used to evaluate side effects derived from caffeine ingestion [11]. This survey also included a specific question to test whether the participants had correctly identified the order of the experimental trials with the purpose of evaluating the success of the blinding procedure.

### Statistical analysis

Data analysis was performed using the SPSS v 20.0 software (SPSS Inc., Chicago, IL). Firstly, the Shapiro-Wilk test was used to test the normality of each variable. All the variables showed a normal distribution (*p*>0.05) and two-way ANOVAs (experimental treatment x genotype group) with repeated measures were performed for all outcome variables to analyze the effects produced by the ingestion of caffeine in AA homozygotes and C-allele carriers. The Bonferroni multiple comparison test was performed to account for multiple comparisons. The magnitude of Cohen’s effect size was calculated for pairwise comparisons. The McNemar test was used to detect differences in the frequencies of side effects reported after the ingestion of each treatment. The results are presented as mean ± SD for each genotype group, and the significance level was set at *p*<0.05.

## Results

There were no differences for age or anthropometric characteristics between AA homozygotes and C-allele carriers (*p* > 0.05; Table 1). The order of the experimental trials was correctly identified by 40% (4 out of 10) of the AA homozygotes and by 44% (4 out of 9) of the C-allele carriers, suggesting the success of the blinding procedure employed for the investigation.

**Fig 1:**
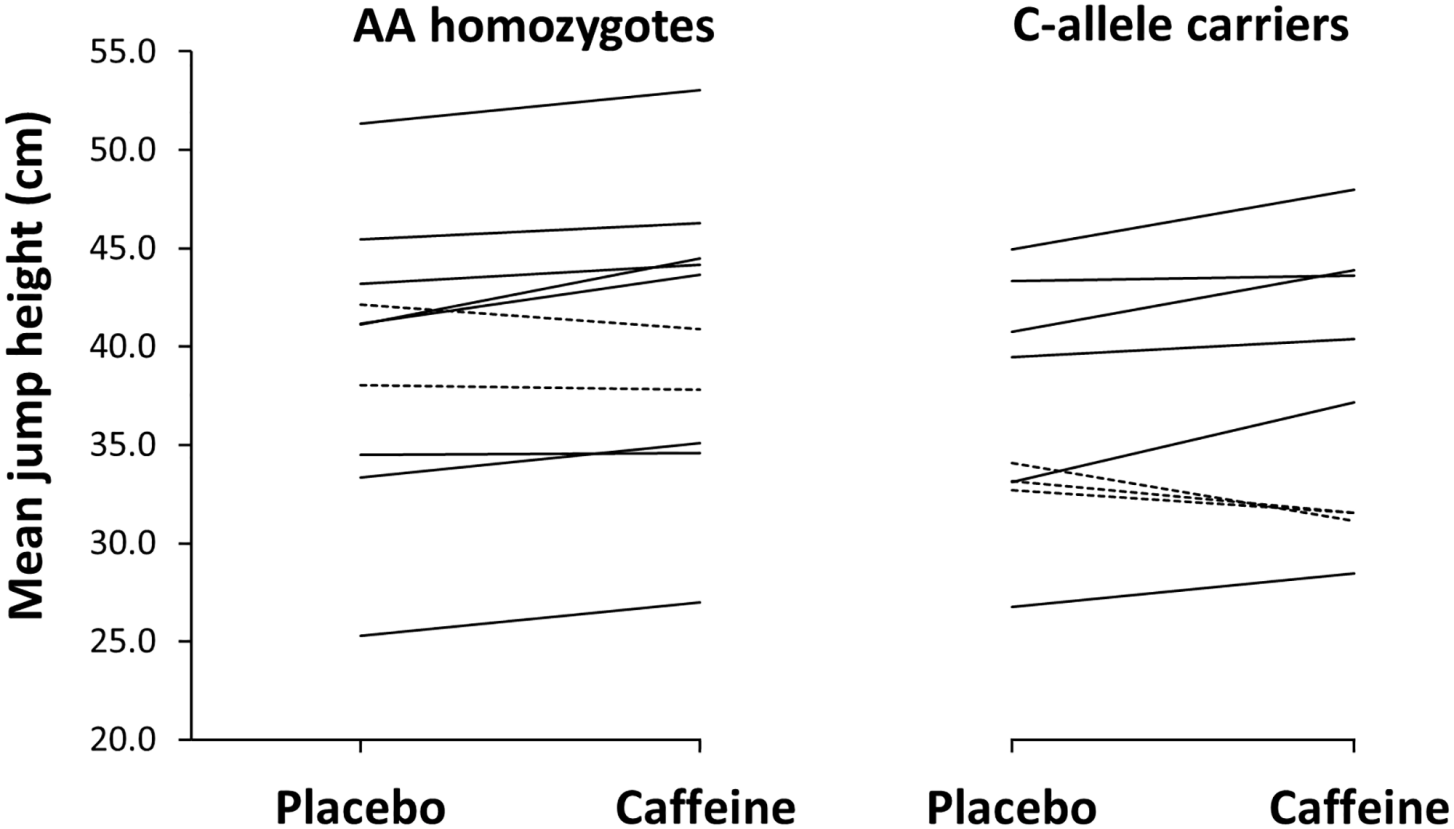
Individual changes in mean jump height during 10 repetitions of the Abalakov jump with the ingestion of caffeine or a placebo. Data are mean ± SD for AA homozygotes (n = 10) and C-allele carriers (n = 9) in the -163C>A polymorphism of the CYP1A2 gene. Note: solid lines represent individuals with an increase in mean jump height with the ingestion of caffeine vs placebo. Dashed lines represent individuals with a decrease in mean jump height with the ingestion of caffeine placebo.

In the 10 repetitions, caffeine increased Abalakov jump height by a mean of 2.9 ± 3.6% in AA homozygotes (*d* = 0.2, *p* = 0.03) while this effect was not significant for C-allele carriers (2.3 ± 6.8%; *d* = 0.2, *p* = 0.33; Table 2). However, there was no interaction between genotypes and treatment for jump height. Specifically, 80% (8 out of 10) of AA homozygotes increased mean jump height with the ingestion of caffeine vs placebo while a similar proportion of C-allele carriers (67%; 6 out of 9) increased mean jump height in the experimental trial with caffeine vs placebo (Fig 1). During the repetitions of the CODAT test without the ball, caffeine did not affect sprint time in AA homozygotes (-0.5 ± 4.0%; *d* = 0.1, *p* = 0.36) or C-allele carriers (0.4 ± 4.0%; *d* = 0.1, *p* = 0.37). Neither did caffeine affect sprint time in the repetitions of the CODAT test with the ball in AA homozygotes (-1.0 ± 4.0%; *d* = 0.4, *p* = 0.15) or C-allele carriers (0.0 ± 3.0%; *d* = 0.0, *p* = 0.49).

**Table 2:** Performance variables during basketball-specific tests and exercise heart rate and total number of body impacts during a 20-min simulated basketball game with the ingestion of caffeine or a placebo. Data are mean ± SD for AA homozygotes (n = 10) and C-allele carriers (n = 9) in the -163C>A polymorphism of the CYP1A2 gene.

Variable	Placebo	Caffeine	Δ (%)	p-value	Effect size
**Mean jump height (cm)**					
AA homozygotes	39.6 ± 7.2	40.7 ± 7.3*	+2.9 ± 3.6	0.03	0.2
C-allele carriers	36.3 ± 5.9	37.2 ± 6.9	+2.3 ± 6.8	0.33	0.2
**CODAT without the ball (s)**					
AA homozygotes	5.91 ± 0.25	5.88 ± 0.27	-0.5 ± 4.0	0.36	0.1
C-allele carriers	5.95 ± 0.33	5.97 ± 0.38	+0.4 ± 4.0	0.37	0.1
**CODAT with the ball (s)**					
AA homozygotes	6.19 ± 0.21	6.09 ± 0.24	-1.0 ± 4.0	0.15	0.4
C-allele carriers	6.14 ± 0.35	6.14 ± 0.41	+0.0 ± 3.0	0.49	0.0
**Mean heart rate (bpm)**					
AA homozygotes	158 ± 9	160 ± 10	+1.3 ± 7.5	0.72	0.3
C-allele carriers	161 ± 13	163 ± 9	+1.2 ± 6.4	0.82	0.1
**Peak heart rate (bpm)**					
AA homozygotes	187 ± 12	188 ± 13	+0.5 ± 4.7	0.22	0.2
C-allele carriers	182 ± 7	185 ± 6	+1.6 ± 3.5	0.46	0.4
**Body impacts (number/min)**					
AA homozygotes	385 ± 48	401 ± 36*	+4.1 ± 5.3	0.02	0.3
C-allele carriers	401 ± 36	415 ± 35*	+3.3 ± 3.2	0.01	0.4

(*) Different from placebo (p<0.05).

**Fig 2:**
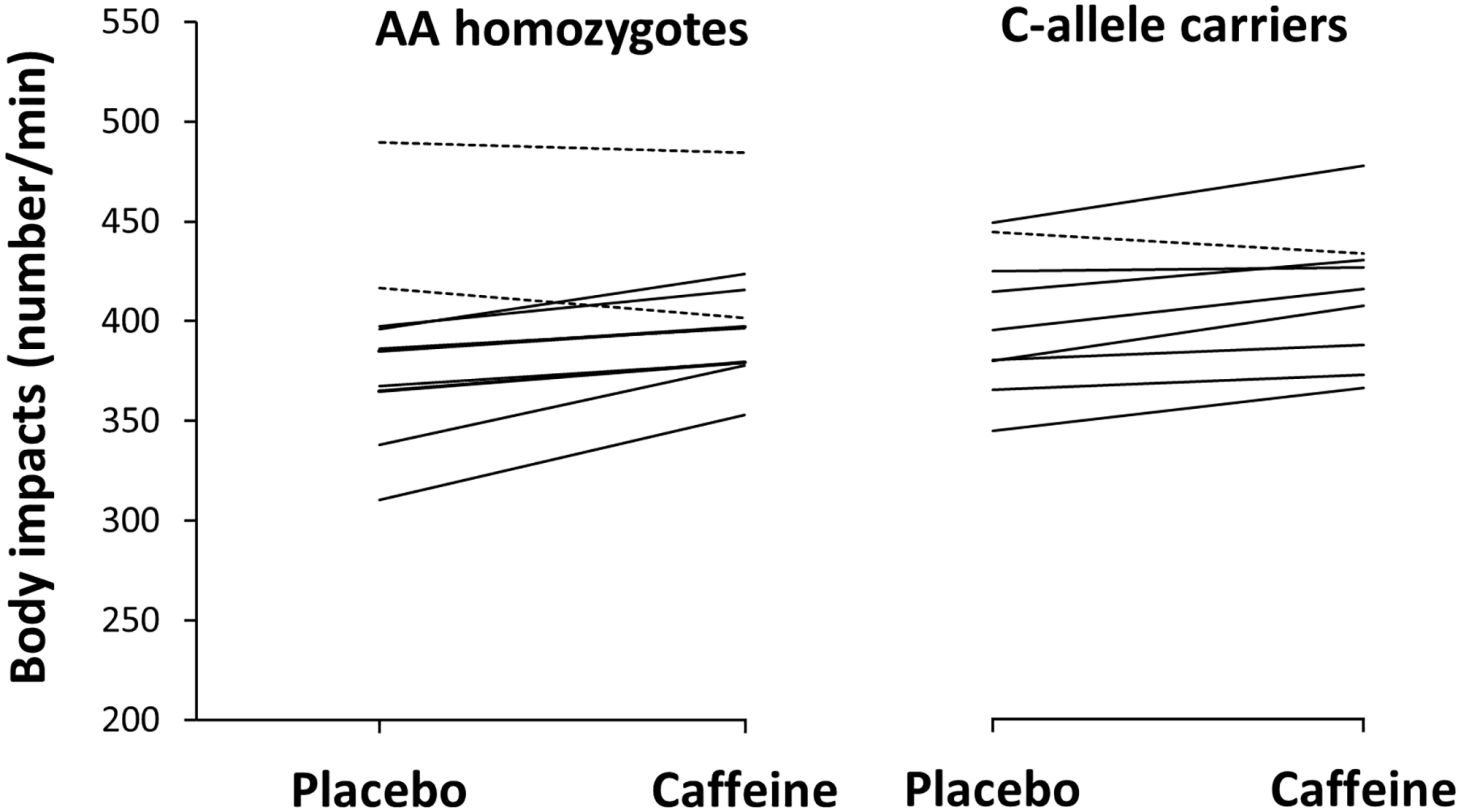
Individual changes in the number of body impacts during a 20-min simulated basketball game with the ingestion of caffeine or a placebo. Data are mean ± SD for AA homozygotes (n = 10) and C-allele carriers (n = 9) in the -163C>A polymorphism of the CYP1A2 gene. Note: solid lines represent individuals with an increase in the number of body impacts with the ingestion of caffeine vs placebo. Dashed lines represent individuals with a decrease in the number of body impacts with the ingestion of caffeine vs placebo.

Caffeine did not significantly modify mean and peak heart rate during the simulated basketball game in AA homozygotes or C-allele carriers (*d* < 0.4, *p* >0.05 for both groups). Nevertheless, AA homozygotes (4.1 ± 5.3%; *d* = 0.2, *p* = 0.02) and C-allele carriers (3.3 ± 3.2%; *d* = 0.4, *p* = 0.01) similarly increased the number of impacts performed during the simulated game. There was no interaction between genotypes and treatment for the number of body impacts during the game while the proportion of basketball players that increased the number of body impacts during the game with caffeine was 80% (8 out of 10) for AA homozygotes and 89% (8 out of 9) for C-allele carriers (Fig 2).

**Fig 3:**
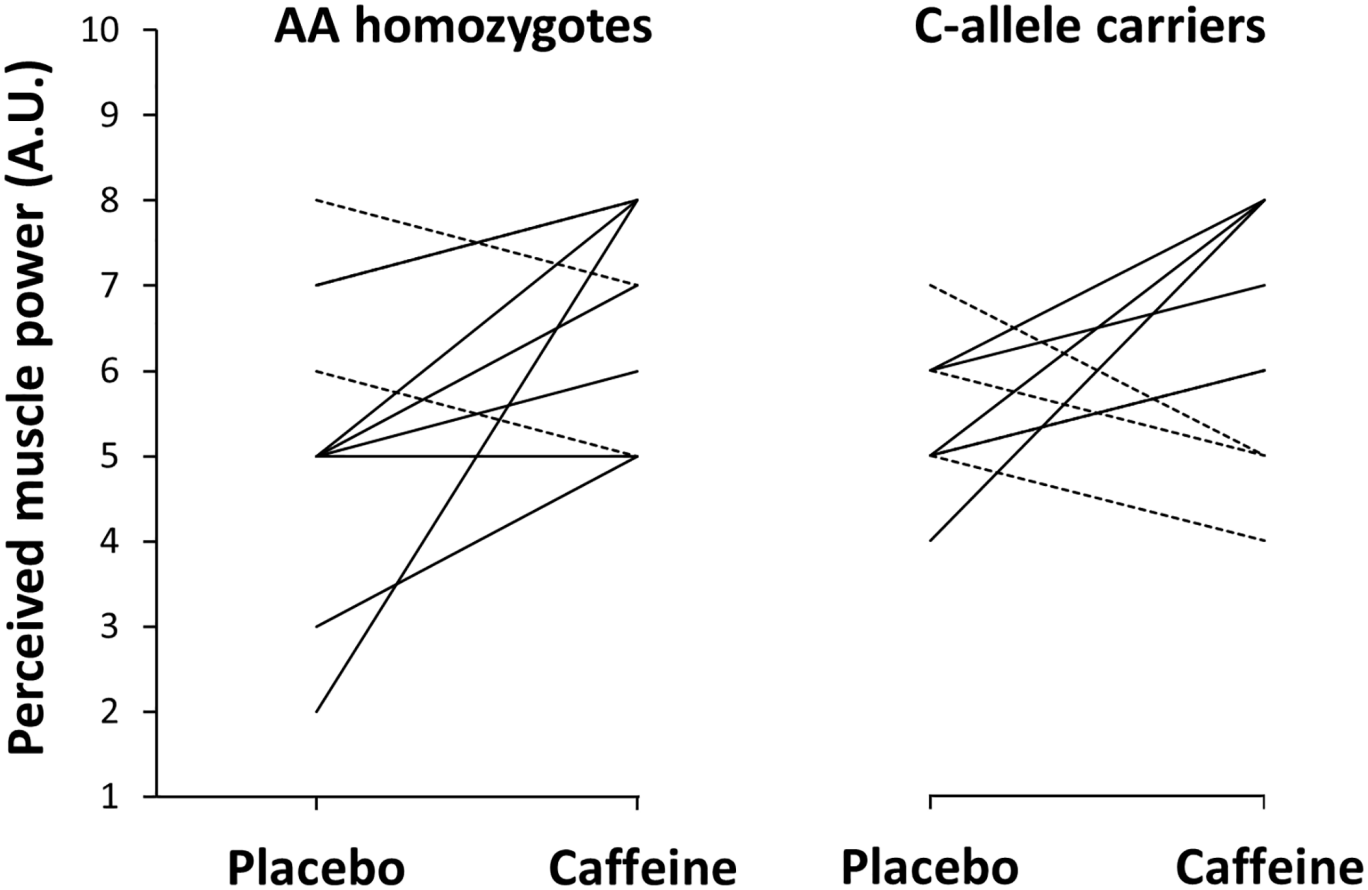
Individual changes for self-perceived muscle power during the basketball-specific testing protocol with the ingestion of caffeine or a placebo. Data are mean ± SD for AA homozygotes (n = 10) and C-allele carriers (n = 9) in the -163C>A polymorphism of the CYP1A2 gene. Note: solid lines represent individuals with an increase in self-perceived muscle power with the ingestion of caffeine vs placebo. Dashed lines represent individuals with a decrease in self-perceived muscle power with the ingestion of caffeine vs placebo.

In comparison to the placebo, AA homozygotes increased the self-perceived muscle power with caffeine by 26.4 ± 11.0% (*d* = 0.8, *p* = 0.04; Table 3). Self-perceived muscle power was also increased by 14.3 ± 11.0% in C-allele carriers although the increase over the placebo did not reach statistical significance (*d* = 0.9, *p* = 0.16). In any case, there was no interaction between genotype groups and treatment for self-perceived muscle power while the proportion of basketball players that reported a greater perception of muscle power with caffeine was similar in the group of AA homozygotes (80%; 8 out of 10) and in the group of C-allele carriers (67%; 6 out of 9; (Fig 3). AA homozygotes tended to increase the ratings of self-perceived endurance capacity with caffeine by 19.3 ± 5.8% (*d* = 0.7, *p* = 0.06) while this variable was unchanged with caffeine intake in the C-allele carriers group (0.0 ± 6.0%; *d* = 0.0, *p* = 0.50). However, there was no interaction between genotype and treatment for self-perceived endurance capacity. The intake of caffeine did not affect the ratings of perceived fatigue in AA homozygotes (d = 0.4, p = 0.20) nor in C-allele carriers (d = 0.0; *p* = 0.50) with no interaction between genotype and treatment for this variable (Table 3). During the 24 h following the experimental trials, AA homozygotes tended to experience increased insomnia with caffeine (Table 4) while C-allele carriers did not present this effect. The remaining side effects were not significantly affected by caffeine intake and they were of a similar magnitude in AA homozygotes and C-allele carriers (*p* > 0.05; Table 4).

**Table 3:** Self-perceived ratings of performance and exertion during a basketball-specific testing protocol with the ingestion of caffeine or a placebo. Data are mean ± SD for AA homozygotes (n = 10) and C-allele carriers (n = 9) in the -163C>A polymorphism of the CYP1A2 gene.

Variable	Placebo	Caffeine	Δ (%)	p-value	Effect size
**Perceived muscle power (A.U.)**					
AA homozygotes	5.3 ± 1.8	6.7 ± 1.3*	+26.4 ± 11.0	0.04	0.8
C-allele carriers	5.4 ± 0.9	6.2 ± 1.5	+14.3 ± 11.0	0.16	0.9
**Perceived endurance (A.U.)**					
AA homozygotes	5.7 ± 1.6	6.8 ± 1.5	+19.3 ± 5.8	0.06	0.7
C-allele carriers	5.6 ± 0.9	5.6 ± 1.7	+0.0 ± 6.0	0.50	0.0
**Perceived exertion (A.U.)**					
AA homozygotes	5.3 ± 1.6	4.6 ± 1.5	-13.2 ± 4.4	0.20	0.4
C-allele carriers	5.4 ± 1.5	5.4 ± 1.5	+0.0 ± 4.4	0.50	0.0

(*) Different from placebo (p<0.05). A.U. = arbitrary units

**Table 4:** Prevalence of side-effects in basketball players 24-h after the ingestion of caffeine or a placebo. Data are frequencies for AA homozygotes (n = 10) and C-allele carriers (n = 9) in the -163C>A polymorphism of the CYP1A2 gene.

Variable	Placebo	Caffeine	p-value
**Nervousness (%)**			
AA homozygotes	10	20	1.00
C-allele carriers	11	0	1.00
**Insomnia (%)**			
AA homozygotes	20	70	0.06
C-allele carriers	22	33	1.00
**Gastrointestinal complaints (%)**			
AA homozygotes	0	20	1.00
C-allele carriers	0	0	1.00
**Activeness (%)**			
AA homozygotes	20	30	1.00
C-allele carriers	11	11	1.00
**Muscle pain (%)**			
AA homozygotes	30	10	0.63
C-allele carriers	11	11	1.00
**Headache (%)**			
AA homozygotes	10	0	1.00
C-allele carriers	11	0	1.00

## Discussion

The aim of this research protocol was to analyze the influence of genetic variations of the CYP1A2 gene on the ergogenic effects conferred by the intake of a moderate dose of caffeine on basketball performance. For this purpose, we analyzed the -163C>A SNP of this gene in a group of 19 experienced basketball players that carried out specific testing and played a simulated basketball game with and without caffeine. According to player’s genotype and previous investigations on this topic [19, 22, 23], two genotype groups were established: AA homozygotes and C-allele carriers. This grouping was based on the higher CYP1A2 enzyme activity found in AA homozygotes that produces faster caffeine metabolism [18] and ultimately could lead to a reduced effectiveness of caffeine in terms of ergogenicity.

The outcomes of this investigation reflect that the ergogenic benefit obtained with the intake of 3 mg·kg^-1^ of caffeine was of similar magnitude for both AA homozygotes and C-allele carriers, with some slight statistical significances between groups but with little biological relevance. For example, AA homozygotes significantly increased mean jump height with caffeine during 10 non-consecutive Abalakov jumps (Table 2). The intake of caffeine did not significantly increase mean jump height in C-allele carriers but the percentage of change induced by caffeine ingestion and the effect size was comparable between AA homozygotes and C-allele carriers (Table 2). In addition, the percentage of basketball players that increased mean jump height with caffeine and the proportion of players that did not obtain benefit from caffeine in this variable were similar in both genotype groups (Fig 1). In the same way, AA homozygotes significantly increased self-perceived muscle power with caffeine. Still, the change in self-perceived muscle power of C-allele carriers after caffeine intake was non-significant when compared to the placebo trial but of the same magnitude as the one found in AA homozygotes. Again, the analysis of the individual responses identified a similar proportion of players that perceived a higher muscle power with caffeine in comparison to the placebo in both AA homozygotes and C-allele carriers (Fig 3). Caffeine also increased the number of impacts during the simulated basketball game while the magnitude of this benefit and the proportions of players that improved the number of impacts with caffeine were very similar for both AA homozygotes and C-allele carriers (Table 2 and Fig 2). This means that basketball players not only increased jump performance and their feelings of muscle power with caffeine, they also were more active during a simulated game which can be translated into improved basketball performance, as recently suggested [10]. These ergogenic benefits were equally present in the group of basketball players with AA homozygosity and the group of players that carried the C-allele for the -163C>A SNP of the CYP1A2 gene, suggesting that this polymorphism is not the responsible for the inter-individual variability in the ergogenic effects found after acute caffeine administration.

The analysis of the questionnaires filled out after each experimental trial reflects that the drawbacks of caffeine ingestion in these basketball players were minimal because the prevalence of side effects were similar between caffeine and placebo trials and very comparable between the genotype groups. Specifically, there was a tendency for increased insomnia in AA homozygotes vs C-allele carriers, and the percentage of AA homozygotes that reported gastrointestinal complains with caffeine was 20% (no C-allele carrier reported gastrointestinal complains with caffeine; Table 4). A recent investigation using the same dosage of caffeine and the same questionnaire to assess caffeine-induced side effects [23] has reported a very comparable frequency of insomnia and gastrointestinal discomforts with the intake of caffeine in AA homozygotes and C-allele carriers after exercise suggesting that the tendency for between-groups differences found in the current investigation might be anecdotical. All this information suggests that caffeine ergogenic effects are equally present in AA and C-allele basketball players, while the downsides are minor in both groups, at least with a dose of 3 mg·kg^-1^ of caffeine. Thus, the CYP1A2 -163C>A SNP exerts minimal influence regarding the benefits and drawbacks obtained by the ingestion of caffeine.

A few previous investigations have tried to determine the genetic influence of the CYP1A2 gene (-163C>A SNP) on the ergogenic effects derived from caffeine intake in athletes or active people [19–23] using similar samples and proportions for the genetic variants of this SNP. According to these investigations, it is unfeasible to draw conclusions about the role of the -163C>A SNP on caffeine ergogenicity because a positive [19], negative [20] and neutral [21–23] ergogenic effect has been found for AA individuals *vs* CA+CC individuals in different cycling trials. Our data suggest that caffeine was equally effective for both AA and C-allele individuals to increase some aspects of their basketball performance, which coincides with the lack of influence of the -163C>A SNP found in previous investigations. Still, further research with larger study samples is needed to ground this notion in order to generalize it to other sport disciplines.

Although we did not discover an association between the CYP1A2 genotype and the extent of the ergogenicity obtained from caffeine intake, previous research is solid regarding the presence of caffeine “non-responders” [13, 14, 29, 30]. We speculate that other genes or other gene combinations could be responsible of the inter-individual difference in response to caffeine ingestion. As the ergogenic effect of caffeine has been mainly related to blockage of the “fatiguing” action of adenosine on its receptors [31], variations of the ADORA2A gene could also be proposed as an explanation for the individual ergogenicity of this substance, mainly because this gene produces different sensitivity to caffeine effects on sleep, anxiety and cognitive performance [32–34]. Only one pilot study has determined a higher ergogenic response after 5 mg/kg of caffeine intake in TT homozygotes for the 1976C>T polymorphism, at least when compared to C-allele carriers in this SNP [35]. However, additional research is required to replicate these findings and to determine other possible genotypes associated to increased/decreased ergogenic response to caffeine intake.

Nonetheless, other non-genetic variables such as individual’s training status, previous habituation/tolerance to caffeine [15] and correct identification of the substance ingested [12] can also contribute to the variability in the ergogenic response to caffeine. In our experiment, the physical fitness of all individuals was high and very similar between groups, as they routinely trained to compete in professional and semiprofessional basketball leagues. All participants were light caffeine consumers and avoided caffeine-containing products for 48 h before testing to reduce the effects of caffeine habituation on the results of this investigation. In addition, participants were asked to guess the order of the trials to determine whether they have identified the trial with the ingestion of the active supplement (e.g., caffeine). However, the percentage of participants that correctly guessed the order of the trials was < 50% in both genotype groups and the players that identified the trial with caffeine did not obtain further benefits when compared to players that did not identify this substance. Even though all these standardizations were carefully set in the experimental procedures, there were some individuals that did not increase their physical performance with caffeine (Figs 1, 2 and 3) suggesting that other factors can contribute to the lack of ergogenic effects after acute caffeine intake. As it has been recently proposed, the use of the term caffeine “non-responder” should be used with caution to differentiate individuals that did not respond to caffeine vs caffeine “non-responders” [15], as this “non-response” may not be present in some individuals when the intervention is repeated.

In summary, AA homozygotes and C-allele carriers for the CYP1A2 -163C>A SNP obtained similar benefits during specific testing and a simulated basketball game from the ingestion of 3 mg·kg^-1^ of caffeine. Although only AA homozygotes significantly improved jump performance and reported higher muscle-power feelings over the placebo trial, a more profound analysis of the effect sizes reveals that C-allele carriers obtained ergogenic effects of a similar magnitude. Thus, the genetic variations of the CYP1A2 gene did not affect the ergogenicity or side effects derived from the consumption of a moderate dose of caffeine. Additional research is necessary to provide an explanation for the inter-individual effects of caffeine intake in sports, specially to explain the nature of non-responders to caffeine.

## Supporting information

S1 Dataset Minimal data set. (XLSX)
